# Roles of gut metabolites on bone mineralization and remodeling: impact of metabolic dysregulation on the gut-immune-bone axis

**DOI:** 10.3389/fendo.2026.1774824

**Published:** 2026-04-28

**Authors:** Sanjay Basak, Kota Sri Naga Hridayanka, Asim K. Duttaroy

**Affiliations:** 1 Indian Council of Medical Research (ICMR) - National Institute of Nutrition, Hyderabad, India; 2 University of Oslo, Oslo, Norway

**Keywords:** bone mineralization, gut dysbiosis, gut metabolites, immunity, postbiotics

## Abstract

Gut microbiota, the community of microorganisms in the gastrointestinal (GI) tract, plays many critical roles in the maintenance of an individual’s health by regulating bone metabolism. Bone mass has been linked to the GI system because nutrients (minerals) are absorbed to maintain bone homeostasis. Dysbiosis of gut microbiota due to metabolic deregulation associated with obesity, fatty liver, and inflammatory status of an individual promotes altered gut microbial diversity, gut-epithelial integrity, releases of harmful gut metabolites those could mediate malabsorption of nutrients such as calcium, vitamin D, omega-3 fatty acids, phosphorous, magnesium, and other key minerals essential for bone mineralization and remodeling in maintaining bone mass through the controlled processes of bone formation and resorption activities. Postbiotics and related products show promising roles in restoring intestinal barrier integrity by reducing systemic inflammation and stimulating the immune system, thereby preventing bone loss. Manipulating the host gut microbiota by sustained release of microbiota-derived metabolites at target sites can help modulate gut homeostasis by regulating redox balance, macrophage polarization, and controlling systemic inflammation. Although several reviews highlighted the roles of gut microbiota, the specific roles of gut metabolites in bone metabolism and associated disorders as a result of metabolic dysregulation remain limited. This narrative review highlights the emerging roles of gut metabolites, postbiotics and related products in regulating intestinal integrity, immune responses, and inflammation, thereby protecting against bone loss.

## 1 Introduction

Metabolic dysregulation involves significant alterations in normal metabolic processes and is a key factor in the development of pathological changes. Dysregulation manifests as lipid abnormalities in obesity, insulin resistance associated with type 2 diabetes mellitus (T2DM), hepatic steatosis, and chronic low-grade inflammation, all of which have a multifaceted impact on bone homeostasis. Metabolic dysregulation negatively affects the gut and immune system, thereby triggering several bone disorders. Recent evidence has highlighted the coexistence of obesity or T2DM with various bone disorders (1, 2). Excess body weight may cause compromised bone quality (3). High-resolution metabolomics has identified that spinal bone mineral density is associated with several metabolic pathways, including fatty acid activation and biosynthesis, glycerophospholipid metabolism, and linoleic acid metabolism (4). A cross-sectional study in postmenopausal women with diabetes indicates altered bone microarchitecture and a negative correlation of bone markers (bone mineral density (BMD), trabecular bone score) with HbA1c levels (5). High levels of bone turnover markers were associated with weak bones and inadequate osteoblastic activity in children with type 1 diabetes (6). Underlying mechanisms such as dyslipidemia, an inflammatory state, and insulin resistance contribute to metabolic dysfunction associated with steatotic liver disease (MASLD), accelerate bone loss, and reduce bone mineral density (7). In addition to the associations between osteoporosis and MASLD, the gut microbiome is a significant factor in their comorbidity (7). Moreover, metabolic dysregulation also affects the immune system, both of which are involved in regulating bone homeostasis.

A bidirectional closed-loop relationship exists between gut dysbiosis and obesity, and is characterized by the Firmicutes-to-Bacteroides ratio (8, 9). Gut dysbiosis is also associated with other metabolic conditions. Increased short-chain fatty acids (SCFA) production is observed when the abundance of Firmicutes increases, which has been associated with increased body weight (10). Dysbiosis of the gut also modulates metabolic pathways related to lipid and glucose metabolism, in addition to its effects on intestinal permeability by altering tight junction proteins (11, 12). Distinct gut microbial signatures are associated with different levels of obesity, with decreased microbial diversity and depletion of specific taxa as severity increases (13). It has been shown that gut dysbiosis and altered metabolite production are key contributors to metabolic dysregulation. Short-chain fatty acids are involved in whole-body metabolism with diverse functions as signaling molecules and key regulators of immunity, glucose metabolism, and lipid metabolism (14). Multiple studies have reported that supplementation with SCFAs in either obesity or T2DM has resulted in reduced serum C-reactive protein (CRP), interleukin 6 (IL-6), insulin, fasting blood sugar, postprandial blood sugar, cholesterol, body mass index (BMI), fat mass, and visceral fat (15–18). Diabetic osteoporosis has been associated with altered SCFA and tryptophan metabolism, leading to decreased bone mass (19). Symbiotic gut microbes, such as Bacteroides uniformis, alleviate metabolic dysfunction related to steatohepatitis by promoting bile acid production, particularly 3-succinylated cholic acid (20). A recent systematic review highlighted the role of gut microbiota composition in postmenopausal osteoporosis (PMOP) (21). Metabolomic analysis of postmenopausal women with low BMD revealed changes in the expression of metabolites involved in tryptophan metabolism (22).

Further, significant associations were observed for altered tryptophan metabolism and pain related to hand osteoarthritis (HOA) (23–25). The correlation of tryptophan metabolites with osteochondral destruction manifests pathologically as osteoporosis, osteoarthritis, and rheumatoid arthritis, suggesting their therapeutic potential (26). Alterations in the gut microbiota affect bone mechanoresponsiveness via metabolite modulation, thereby activating a positive feedback loop between nitric oxide and calcium in osteocytes, suggesting a promising role in the development of anti-osteoporotic agents (27). Various studies show that bone mass is regulated by the gut microbiota (28). Earlier, we reported that the gut-bone axis could mediate chondroprotective effects in the amelioration of osteoarthritis (29, 30). The diverse composition of the microbial population and its multitude of functions also modulate the immune system and the bone homeostasis. Despite some evidence, their exact effects remain unclear.

Clinical evidence reveals distinct microbial and metabolic profiles, particularly an enrichment of *Lachnospira eligens* in fracture patients with low bone mass, highlighting the potential pathogenic role of specific microbial species (31). In postmenopausal patients, associations between bone mineral density, gut microbiome, and serum metabolome, specifically SCFAs, show interrelationships linked to bile acid metabolism, calcium absorption, and lipid metabolism (32–34). To give a specific example, Akkermansia muciniphila, a mucin-degrading bacterium, affects bone metabolism by immune modulation, metabolite signaling, or cellular crosstalk between the gut and bone (35). Mendelian randomization also emphasizes the role of specific plasma metabolites in osteoporosis risk (36). Analysis of genetic risk scores of microbial taxa revealed significant associations with osteoporotic fracture risk (37). In rodent studies, microbial profiling revealed distinct metabolomic signatures across different osteoporosis models (38, 39). The microbiota-mediated crosstalk between the gut ecosystem and skeletal system occurs via several signaling pathways, including osteoprotegerin (OPG)/receptor activator of NF-κB, bone morphogenic protein (BMP)/SMAD, OPG/receptor activator, and Wnt pathways by collectively targeting the fate of osteoclasts, osteoblasts, calcium absorption, hormone secretion, and immune responses (40).

**Figure 1:**
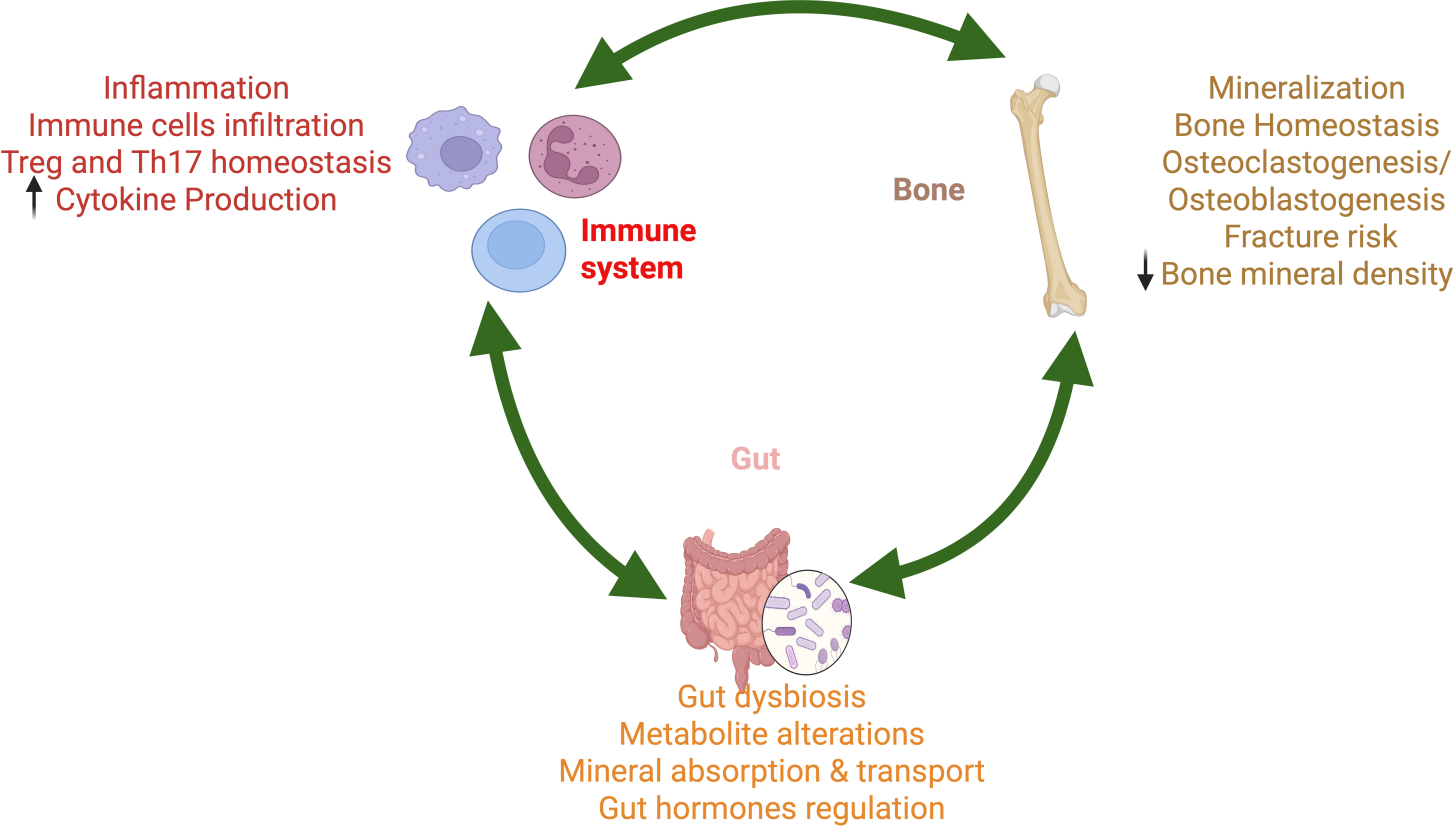
Host metabolic dysregulation disrupts homeostasis of bone, gut, and immune system working via an interconnected feedback loop. In the gut, metabolic dysregulation causes dysbiosis, altered metabolite production, altered mineral absorption and transport, and altered gut hormone production. Metabolic dysregulation results in chronic inflammation, characterized by increased cytokine production, immune cell infiltration, and impaired Treg/Th17 homeostasis. The interconnected feedback loop further exacerbates the underlying mechanisms of bone disorders, including reduced bone mineral density, increased fracture risk, altered mineralization, and imbalances in osteoclastogenesis and osteoblastogenesis.

The gut microbiome has been reported to be involved in immune system interactions at both innate and adaptive levels, including immune cell activation, immune response generation, and immune cell balance (41–43). Gut secreted and mediated metabolites, vitamins, and minerals all play an active role in the initiation of immune response (44). Of late, several pieces of evidence point towards the modulation of a three-way gut-immune-bone axis (45–48). Increased inflammation and reduced SCFAs due to gut dysbiosis aggravate T cell imbalance, synovial inflammation, and bone destruction (49). Dietary-derived propionate, a gut metabolite, has been shown to impair the function of synovial fibroblasts during inflammatory tissue priming in rodent arthritis by modulating the loss of inflammatory and immune-regulatory mediators (50), a process suggested to be dependent on microbial populations that modulate Th17 cells (51). Further, Th17 lymphocytes and receptor activator of NF-kB ligand (RANKL) ligand system both exhibit crucial roles in the gut-immune bone axis in osteoporosis (52). The population of Th17 cells is also modulated by particular Prevotella species (*P. copri* and *P. melaninogenica*) via their interaction with toll-like receptor 2 (TLR2) (53). A recent study integrating single-cell RNA sequencing and mendelian randomization revealed key mediator genes – TAF1A (TATA-box binding protein associated factor 1A), USP6NL (ubiquitin-specific protease 6 N-terminal like), and SELENOT (selenoprotein T) in osteoporosis that regulate pathways related to immune regulation, calcium homeostasis, microbiota modulation, and bone metabolism, suggesting the involvement of the gut-immune-bone axis (54). Diverse effects of metabolic dysregulation on the gut-immune-bone axis are shown in **Figure 1**.

**Figure 2:**
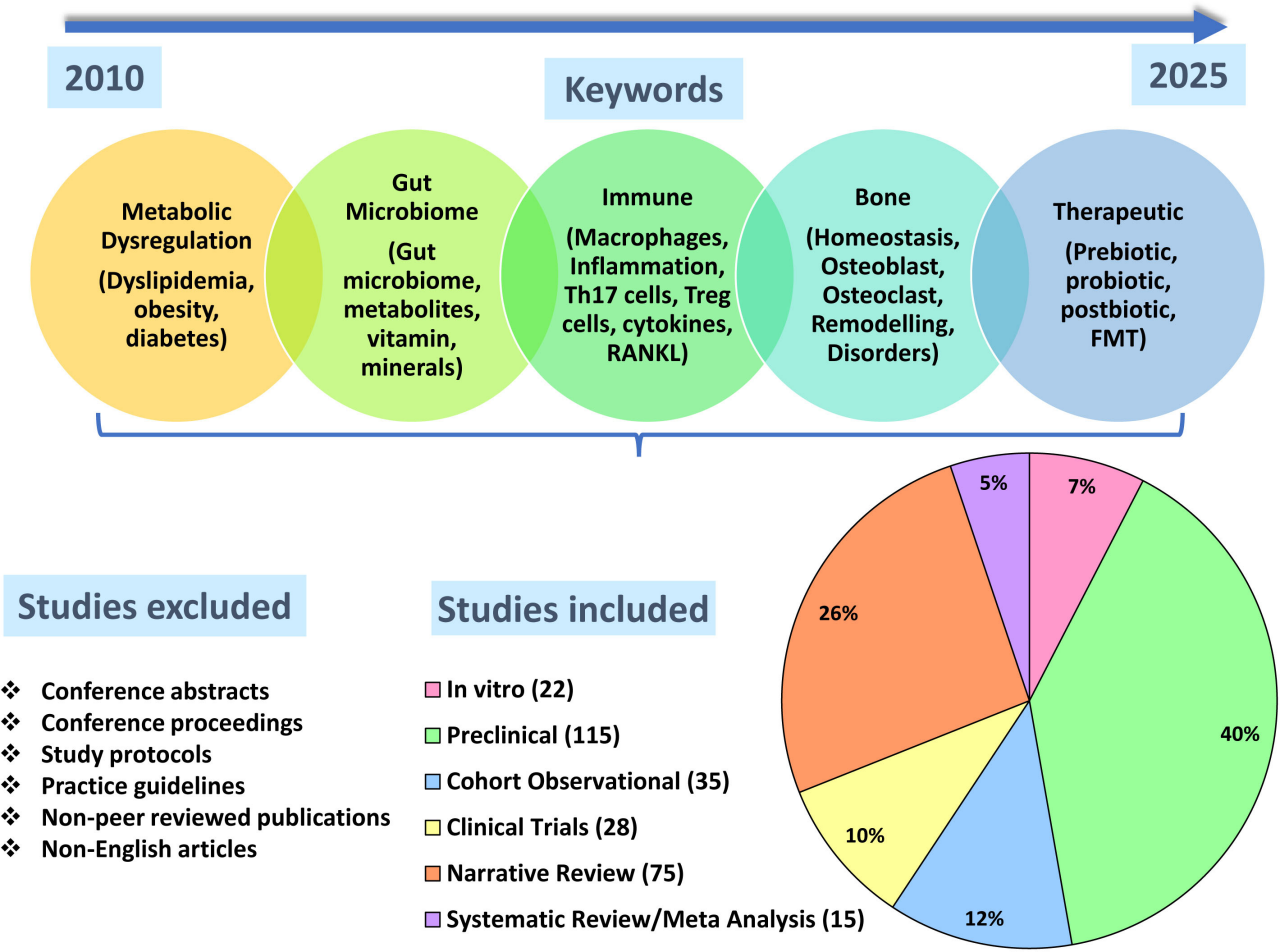
Workflow of the narrative review. Studies from 2010–2025 were included in the review using the mentioned keywords and exclusion criteria. Various study types were included to provide a comprehensive narrative overview. Number of studies included in each category are mentioned in the parentheses while the pie chart depicts their relative proportions as percentage of the total. RANKL, Receptor activator of NF-κB ligand; FMT, Fecal microbiota transplantation.

A thorough, structured and non-systematic review of the literature was conducted between October 2025 and December 2025 using PubMed, Science Direct and Scopus databases using Boolean operators and search terms like “metabolic dysregulation”, “obesity”, “diabetes”, “inflammation”, “bone”, “bone homeostasis”, “immune system”, “T cells”, “osteoarthritis”, “osteoporosis”, “gut metabolites” and other similar keywords. Peer-reviewed published articles over the past 15 years (2010-2025) were included from these searches. The current narrative review includes studies showing mechanistic insights (*in vitro* and pre-clinical), clinical studies, and relevant review articles. Non-peer-reviewed publications, conference abstracts, study protocols, practice guidelines, studies lacking sufficient methodological details, studies not relevant to the scope of the review, and articles not available in English were excluded, as shown in **Figure 2**.

Despite substantial evidence on gut microbiota, which is profoundly involved in maintaining individual bone health, evidence on the contribution of gut metabolites to bone disorders is limited. While earlier reviews have discussed how gut metabolites impact bone health (55), this narrative review places emphasis on the impact of metabolic dysregulation on gut-mediated metabolite synthesis, vitamin production, and mineral absorption. In addition to highlighting bone-immune crosstalk, this review provides a comprehensive overview of current data on therapeutic targeting of the gut-immune-bone axis, which can help restore bone homeostasis in both healthy and disease states.

## 2 Multifaceted influence of the gut on bone health

### 2.1 Gut-mediated metabolite production

The diversity of the gut microbiome shapes the production of gut metabolites that can diffuse to distant organs and regulate cellular mechanisms. Metabolic and skeletal changes accompany compositional and structural alterations in the gut microbiome and in gut-derived metabolites (56, 57). Gut metabolites such as short-chain fatty acids, tryptophan derivatives, bile acids and polyamines are involved in bone remodeling by promoting osteoblastogenesis and inhibiting osteoclastogenesis. In particular, SCFAs encourage bone growth and remodeling and reduce pathological bone loss via induction of IGF-1 (58, 59). While SCFAs originate in the intestinal lumen, several membrane-bound G protein-coupled receptors are involved in their transportation to various peripheral organs. Expression of SCFA receptors GPR41 (free fatty acid receptor 3) and GPR109A (hydroxycarboxylic acid receptor 2) in osteoblast precursors and subsequent inhibition of osteoclast differentiation by propionate and butyrate suggest the potential direct modulation of bone resorption by SCFAs (60). Valeric acid has also been evidenced to promote bone micro-structure and reduce bone resorption (61). SCFAs also mitigate osteoclast-mediated bone remodeling during arthritis, potentially by binding to the free fatty acid receptor 2 (62, 63). Butyrate shows a positive correlation with Muribaculaceae explicitly. It is responsible for gut-bone communication by increasing the number of regulatory T cells, which activate the Wnt pathway for bone formation (64–66).

Gut secreted bile acids have been associated with abnormal bone metabolism. A recent systematic review highlighted postmenopausal osteoporosis exhibiting an increase in bile acids (21). Increased bile acid levels observed in osteoporosis are negatively correlated with bone mineral density (67, 68). Further, elevated bile acid turnover leads to abnormal alterations in lipids and lipid-soluble compounds in osteoporosis (69). Elevated levels of gut-derived circulating polyamines, specifically putrescine and spermidine, in obesity/T2D individuals (70, 71) suggest a positive correlation with obesity and also imply possible protective effects (72, 73). Dysregulated polyamine metabolism impacts lipid, glucose, and energy homeostasis (74, 75). While all polyamines spermine, spermidine, and putrescine promote osteogenic differentiation of mesenchymal stem cells (76), spermidine is explicitly involved in mitigating osteoarthritis and fragility fractures (77, 78). Similarly, another metabolite, urolithin, was found to ameliorate osteoclastogenesis and osteoporosis by attenuating NF-κB signaling (79–81).

Gut microbiota-derived trimethylamine N-oxide (TMAO) has been implicated in adverse effects on bone. Higher levels of TMAO could increase the risk of obesity (82), are associated with risk of hip fractures in obese/overweight individuals (83), low bone mineral density and osteoporotic fractures in T2D patients (84). Mechanistically, excessive TMAO accelerates osteoporosis by promoting loss of bone mass via the inhibition of osteogenic differentiation (85, 86). TMAO also enhances RANK ligand-induced osteoclast differentiation and bone resorption via activation of the NF-κB pathway (87, 88).

Tryptophan and its metabolites show versatile modulatory roles in bone remodeling via the regulation of osteoblasts, osteoclasts, and chondrocytes. Indole acetic acid (IAA) and indole-3-propionic acid (IPA) act as aryl hydrocarbon receptor ligands and promote barrier integrity. These Trp metabolites further increase M2 polarization of macrophages, resulting in extensive release of IL-10 that inhibits osteoclastogenesis and promotes osteoblastogenesis during osteoporosis (89–91). Low levels of indole-3-lactic acid, a tryptophan metabolite is associated with hand osteoarthritis in two independent cohorts (23). Activation of the kynurenine pathway, involved in tryptophan metabolism during osteoblastogenesis and osteogenic differentiation by picolinic acid, suggests their effects in promoting bone formation and bone mass (92, 93).

Furthermore, modulation of the kynurenine pathway by inflammation in conditions like osteoporosis and osteosarcopenia suggests its potential as a disease biomarker (94). Conversely, gut-derived serotonin (TPH1) inhibits bone formation by binding to osteoblasts and reducing osteoblast proliferation (95). Subsequently, inhibiting serotonin biosynthesis with a small-molecule inhibitor mitigates osteoporosis in a dose-dependent manner (96).

### 2.2 Gut mediated vitamin synthesis

Vitamin D is a major regulator of bone homeostasis by promoting calcium absorption (97). Despite the classical synthesis of vitamin D, a bidirectional relationship exists between vitamin D and the gut, influencing both the microbiota and vitamin D status. Epithelial cells in the gut abundantly express the vitamin D receptor (VDR). Gut metabolites are known to modulate VDR expression, thereby strengthening barrier integrity and immune responses by maintaining vitamin D homeostasis (98–100). The significant consequences of vitamin D deficiency are bone loss leading to osteoporosis, mineralization defects, and increased bone turnover, which disrupts bone homeostasis (101). Vitamin D deficiency in murine models also leads to increased weight gain and compromised bone biomechanical properties (102). A recent cross-sectional analysis in minority children and adolescents as part of the MetA-bone trial revealed that overweight/obesity and suboptimal vitamin D status were associated with gut barrier integrity and inflammation (103). Further, an abundance of *Bifidobacterium* was associated with altered cholecalciferol absorption in patients with osteoporosis, thereby influencing disease progression (104). In mice, *B. longum* influenced serum vitamin D metabolite levels and alleviated osteoporosis by modulating the intestinal flora (105). Probiotic supplementation, however, shows promising potential. A randomized controlled trial showed improvements in serum vitamin D3 levels and bone metabolism in PMOP patients upon supplementation with a *B. lactis-*based probiotic (106).

With respect to vitamin B12, gut microbiota in an obesogenic environment has a depleted abundance of enzymes involved in the B12 synthesis pathway, thus reducing the cobalamin levels in obese individuals (107–109). Furthermore, in obesity, vitamin B12 has been associated with both the abundance and diversity of the gut microbiota (110). Vitamin B12 deficiency has been associated with increased BMI and adiposity, thereby affecting individuals’ metabolic health (111, 112). Since vitamin B12 is known to play a crucial role in the remethylation of homocysteine to methionine, its deficiency is also associated with high concentrations of serum homocysteine, thus increasing the risk for fracture incidence (113, 114). Meta-analyses and Mendelian randomization studies reveal associations of homocysteine and subsequent B12 deficiency with PMOP (115) and osteoarthritis (116), respectively. In addition, B12 deficiency has been shown to negatively affect bone mass and bone formation by decreasing taurine synthesis (111, 117, 118). Supplementation with vitamin B12 has been shown to benefit BMD, as evidenced by various randomized controlled trials (119, 120). Supplementation with probiotics and related products has been discussed separately in Section 4.2.

Vitamin K levels are significantly associated with bone metabolism, fracture risk as well as abundance of specific gut microflora – *Bacteroides* and Rikenellaceae (121). In addition, low vitamin K levels are associated with low bone mineral density and subsequent osteoporosis in patients with Crohn’s disease (122). Vitamin K is known to activate the pregnane X receptor (PXR), which has diverse effects on bone, glucose, and lipid metabolism (123, 124). Clostridium has shown similar activity derived from indole propionic acid, which activated PXR and thus reduced osteoclast formation in ovariectomized (OVX) mice (125). Furthermore, in OVX rats, vitamin K2 improved calcium balance and promoted femoral cortical thickness (126). In addition to the independent effects of both vitamin K and vitamin D, preclinical and clinical evidence suggest their synergistic effects on bone remodeling (127–129).

### 2.3 Gut-mediated mineral metabolism and transport

As discussed earlier, calcium plays a crucial role in bone mineralization and is available only from dietary sources. The calcium signaling pathway influences bone resorption and osteoclast activation (130). While calcium homeostasis is regulated by intestinal absorption, bone turnover, and renal reabsorption, its dysregulation affects lipid metabolism and alters bone homeostasis, resulting in fragile bones with increased fracture risk (131, 132). Observational studies, genome-wide association studies, and mendelian randomization studies imply the influence of calcium and phosphate homeostasis in the gut-bone axis (133–135). Calcium deficiency, when accompanied by phosphate deficiency, reduced vertebral strength in rats by reducing trabecular bone volume (136). Several ion channels, including calcium release-activated calcium channels, voltage-sensitive calcium channels, voltage-gated calcium channels, TRP family channels, potassium, and sodium channels, have been reported to be vital in bone mechanotransduction and metabolism (137). The expression of the extracellular calcium-sensing receptor (CaSR) has been reported in osteoblasts and chondrocytes, wherein it mediates the transdifferentiation of chondrocytes into the osteoblastic lineage (138–141). In addition to calciotropic hormones such as parathyroid hormone and calcitonin that regulate intestinal calcium transport, FGF-23 (fibroblast growth factor-23) also exerts inhibitory effects (142). In this regard, CaSR inhibitors could suppress FGF23 upregulation, suggesting that the resultant inhibition of calcium transport by FGF23 occurs under conditions of excess calcium (143). Due to its function as a vital signaling molecule, calcium interacts with several enzymes in the body. Knockout of intestinal alkaline phosphatase (IAP) in mice resulted in significantly higher calcium absorption and improved trabecular bone properties, suggesting a negative correlation between intestinal calcium uptake and IAP activity (144). Involvement of voltage-gated calcium channels in bone microdamage indicates a role for intracellular calcium signaling (145, 146).

Inorganic phosphate, a vital constituent of cells, is essential for mineralization and bone maintenance, with 80% existing as hydroxyapatite (calcium phosphate crystals) (147). Phosphate absorption in the intestine occurs either via the paracellular route across the concentration gradient or the transcellular route through sodium-phosphate cotransporters (148, 149). Intestinal inflammation also negatively affects calcium and phosphate transport, leading to bone loss (150). Disruption of phosphate homeostasis results in either hypophosphatemia, leading to mineralization defects, or hyperphosphatemia, leading to excessive bone ossification (151). In this regard, a subset of hormone-like proteins called phosphatonins, such as FGF-23, FGF-7, and matrix extracellular phosphoglycoproteins, have been implicated in phosphate absorption and, consequently, in hypophosphatemic rickets and osteomalacia (148). In addition to phosphatonins, Vitamin D3 and the parathyroid hormone also regulate phosphate in the skeleton, gut and kidneys (152). However, a significant involvement of FGF-23 derived from osteoclasts and osteoblasts suggests its role in the gut-bone axis and prompts towards anti-FGF23 therapeutics (153, 154).

**Figure 3:**
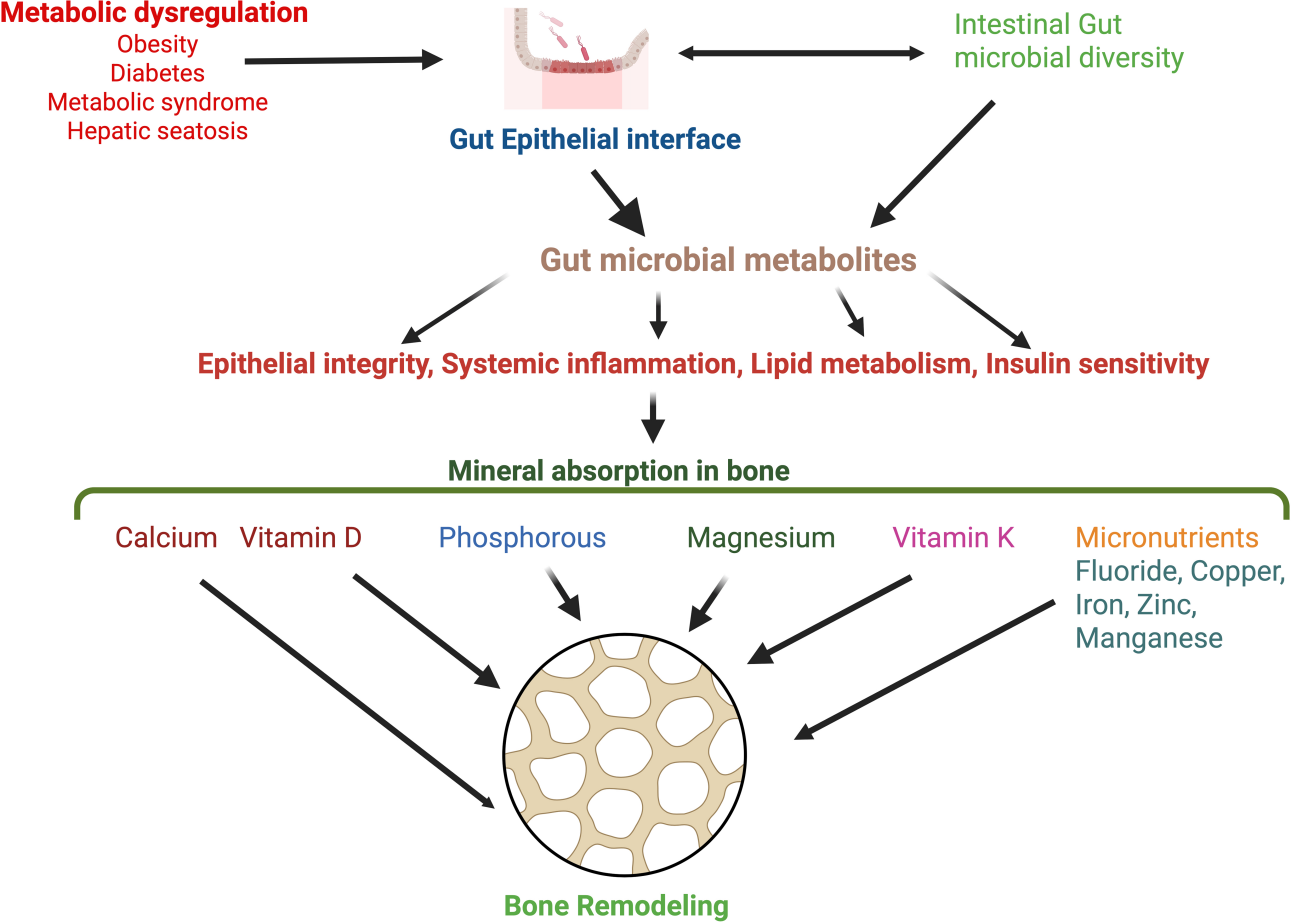
Metabolic dysregulation and associated disorders, including metabolic syndrome, diabetes, obesity, and hepatic steatosis, may modulate the gut epithelial interface and exert effects on gut microbial diversity and metabolite production. Altered gut metabolites can influence mineral absorption in bone through host metabolic and inflammatory responses. While calcium and phosphorus are the major mineral components of the bone, other minor minerals like magnesium, zinc, iron, copper, and manganese act as vital micronutrients during bone remodeling. Distinct roles of vitamin D and vitamin K contribute to bone homeostasis. Alterations in gut metabolites reduce vitamin K synthesis and impair vitamin D activation, thereby affecting bone remodeling.

Despite little emphasis on micronutrient levels and absorption, trace elements exert a positive influence on bone health, with deficiencies leading to accelerated bone loss, deterioration of bone quality, and an increased risk of fractures (155). Since the gut microbiome actively regulates the transport and metabolism of micronutrients, their deficiencies promote microbial imbalance and contribute to metabolic dysregulation (156, 157). Zinc, an essential mineral, appears to be involved in bone regeneration by modulating osteoclast and osteoblast functions (158, 159). Zinc transporters play a crucial role in the absorption of dietary zinc, with zinc metabolism occurring primarily in the bone, gut, erythrocytes, and muscle (160). Manganese is a crucial cofactor for several enzymes and is involved in bone remodeling. Manganese induces osteoblast proliferation, inhibits osteoclastogenesis, and preserves bone mass via the RANK/RANKL/OPG pathway (161). Mn levels are primarily governed by intestinal absorption and are regulated by interactions with other nutrients, such as iron (162). However, increased levels of Mn are linked to phosphate depletion, causing rachitic lesions and altered endochondral ossification (163). Evidence also suggests interactions between environmental fluoride exposure and the microbiota, leading to altered microbial and SCFA composition, reduced barrier integrity, and inflammation, thereby influencing bone metabolism (164). Further, a calcium-deficient and phosphate-deficient diet, when combined with high fluoride, was shown to reduce vertebral bone strength and impair bone mineralization in rats (136). An imbalance in iron regulation is also associated with an altered microbiome and impaired glucose metabolism, leading to insulin resistance (165). Dysregulation of copper homeostasis has implications for the pathophysiology of several bone diseases, such as osteoarthritis, RA, and osteosarcoma (166). Various roles of gut metabolites on mineral absorption and bone remodeling are highlighted in **Figure 3**.

## 3 Immune and cytokine milieu

### 3.1 Osteocyte/osteoblast crosstalk with T lymphocytes

Bone remodeling and homeostasis are maintained by osteoclasts and osteoblasts, which mediate bone resorption and bone formation, respectively.

T lymphocytes exhibit both stimulatory and inhibitory effects on bone resorption (167). In particular, Th17 and Th9 cells secrete cytokines IL-17, IL-9, and TNF-α that induce RANKL expression, stimulate osteoclastogenesis, and promote bone resorption (168, 169). However, Th1, Th2 subsets of T lymphocytes and Treg cells inhibit osteoclastogenesis via IFN-γ, IL-4, IL-12, IL-13, and IL-18, thus reducing bone resorption (170–172). CD8 T cells form a negative feedback loop with osteoclasts, contributing towards both skeletal and immune homeostasis (173, 174). In PMOP, a specific subset of T cell population defined as CD8a + GZMK + T cells was evidenced to inhibit osteoclastogenesis via the p38-MAPK pathway (175). While osteoclast progenitors are directly involved, osteoblasts also contribute towards osteoclastogenesis via cell-to-cell contact with osteoclast progenitors. CXCL9, a chemokine secreted from osteoblasts and involved in the recruitment of T cells, aggravates osteoporosis by positively regulating osteoclasts, stimulating bone resorption, and inhibiting osteogenesis (176). Both synovial fibroblasts and T lymphocytes produce several cytokines, including RANKL, Interferon γ (IFN-γ), Interleukin 17 (IL-17), and granulocyte-macrophage colony-stimulating factor, that regulate osteoclastogenesis (177, 178). Given the role of IL-17 in promoting osteoclast differentiation and subsequent bone loss, recent evidence suggests a glutamine-dependent regulation of IL-17, highlighting the potential functions of the immune-metabolic axis (179). IL-9 from Th9 cells shows pathological effects in inflammatory bone loss during PMOP and RA by enhancing osteoclastogenesis (180, 181). Osteoclasts emerge as immune-competent cells, as they secrete high levels of T cell chemoattractants and suppress T cell proliferation (182). Inflammatory osteoclasts identified by CX_3_CR1 stimulate TNF-α-producing CD4+ T cells via IL-17, demonstrating their role in bone destruction (183). Further in TNF-α-producing CD4+ T cells, NOD-like receptor family C (NLRC) could attenuate Th17-dependent osteoclastogenesis by reducing macrophage inflammatory protein (MIP-1α), macrophage chemoattractant protein (MCP1), RANKL and inhibiting activation of NF-κB (184). Osteoporotic T cell exosomes, isolated ex vivo, altered osteoblastic function by reducing ALP activity and the expression of osteopontin, osteocalcin, Runx2, and type 1 collagen, thus exhibiting potential in bone remodeling (185). Direct contact between T lymphocytes and osteoblasts also enhances matrix mineralization by simultaneously upregulating ICAM-1 and suppressing TGF-β in inflammation-associated bone mineralization (186).

### 3.2 Th17/Treg homeostasis

**Figure 4:**
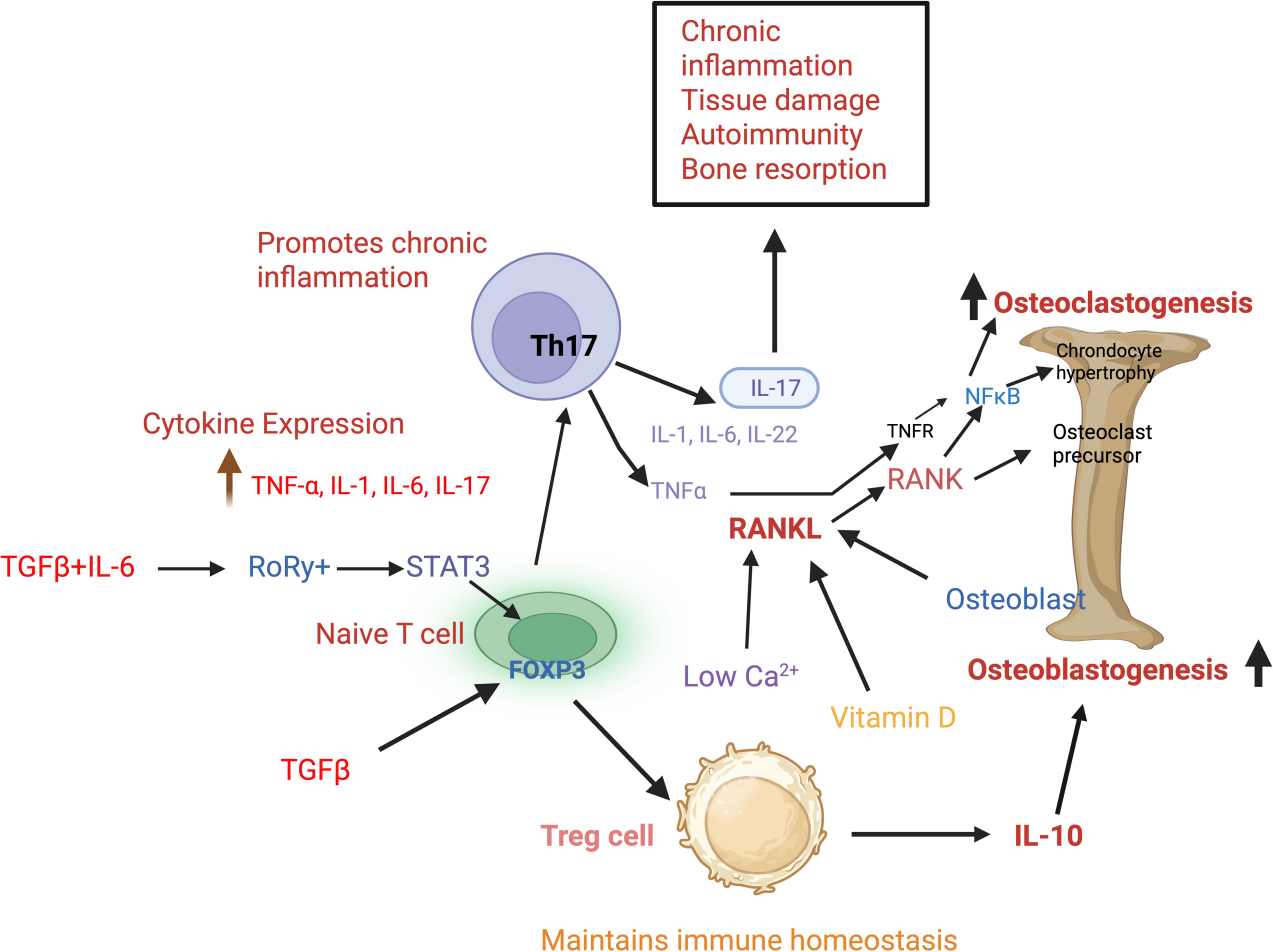
Naïve T cells contribute towards bone homeostasis by maintaining a balance between Th17 and Treg cells. Regulatory T cells suppress the pro-inflammatory microenvironment by secreting IL-10 and TGF-β. While indirectly promoting osteoblastogenesis, Treg cells inhibit inflammation-induced osteoclastogenesis, thus maintaining immune and bone homeostasis. In isolation, TGF-β drives naïve T cells to differentiate into Treg cells via FOXP3, and in the presence of both TGF- β and IL-6, naïve T cells differentiate into Th17 cells via RORγt and STAT3. Th17 cells promote chronic inflammation, bone resorption, and tissue damage by secreting distinct cytokines TNF-α, IL-1, IL-6, IL-17, and IL-22. In addition to osteoblast cells and Th17 cells, low calcium and vitamin D levels also increase RANKL production. RANKL via RANK and NF-κB activates osteoclast precursors and promotes osteoclastogenesis. IL-10, Interleukin 10; TGF-β, Transforming growth factor beta; FOXP3, Forkhead box P3; RORγt, Retinoic acid related orphan receptor gamma t; STAT3, Signal transducer and activator of transcription 3; TNF-α, Tumor necrosis factor alpha; RANKL, Receptor activator of NF-κB ligand; RANK, Receptor activator of NF-κB; NF-κB, Nuclear factor kappa B.

Th17 and Treg balance is governed mainly by the intestinal microflora, metabolic factors, and various signaling pathways (187–189). Despite CD4+ T cells being the common precursors of both Th17 and Treg cells, differentiation into Treg cells requires TGF-β (190, 191). Under different cytokine conditions, TGF-β also contributes to Th17 cell differentiation. In the presence of both TGF-β and IL-6, Foxp3 is inhibited via signal transducer and activator of transcription 3 (STAT3), which induces differentiation of naive T cells into Th17 cells (190). T lymphocyte imbalance also shows pathological significance in Rheumatoid arthritis. In addition to reports suggesting that Th17 cells are generated either locally, by cytokine-mediated mechanisms, or in the gut by commensal bacteria, evidence also points towards the conversion of Foxp3 (+) T cells into pathogenic Th17 cells with a distinct gene expression pattern and potent osteoclastogenic ability (192). Treg cells (CD4^+^CD25^+^Foxp3^+^) inhibit osteoclastogenesis by suppressing RANKL and M-CSF, while Th17 cells secrete TNF-α, IL-1, IL-6, IL-17, and RANKL, which enhance osteoclastogenesis (193, 194). Gut resident regulatory T cells also share similar functions, enhancing osteoblastogenesis and thereby inhibiting osteoclastogenesis during osteoporosis (195). Further, T cells with the Th17 lineage emerge as potent osteoclastogenesis mediators in osteoporosis via the upregulation of IL-17, IL-23, and increased expression of transcription factors RORγt and RORα (169, 196). In osteogenesis imperfecta, recurrent bone fractures are associated with increased secretion of proinflammatory cytokines. Reduced T cell activation, cytokine secretion and enhanced bone remodeling post systemic transplantation of Treg cells suggest their effects in dampening the pro-inflammatory microenvironment (197). In rheumatoid arthritis, Treg cells alleviate inflammatory responses via β2-AR/β-Arr2/ERK signaling (198). Interestingly, the cytokine milieu during various bone disorders also induces Th17 plasticity, wherein factors, including transcription factors and other molecular cues, promote differentiation of Th17 cells into Th17/1 transitional cells and Foxp3+ T cells (199, 200). The influence of Th17 and Treg cells on bone homeostasis is presented in **Figure 4**.

### 3.3 Immunomodulatory effects of gut-derived factors

Interestingly, short-chain fatty acids have been reported to mediate the immunomodulatory activities of regulatory T cells (Treg) and macrophages (201). Involvement of SCFAs in regulating bone mass or inhibiting osteoclastogenesis occurs in a Treg-dependent manner (66). Further, postmenopausal osteoporosis has been associated with a pro-inflammatory state characterized by a lower population of Treg cells and a higher population of Th17 cells (21). Polyamines also show a modulatory effect by supporting the proliferation and differentiation of T cells (202, 203) and maintaining Treg/Th17 homeostasis (204). Further, gut polyamines have ameliorative effects on gut dysbiosis associated with obesity-related T2D and metabolic syndrome, in addition to regulating host immunity (72). Specifically, spermidine reduces the inflammatory microenvironment by promoting Treg proliferation and differentiation and simultaneously decreasing Th17-derived IL-6, IL-17, and TNF-α (77, 78). Glycolithocholic acid, a bile acid metabolite promotes osteogenic differentiation during PMOP by promoting differentiation into Treg cells (205). Trp metabolites, particularly 3-hydroxyanthranilic acid also restore Th17/Treg homeostasis (206). Calcitriol (1,25-dihydroxycholecalciferol) has been shown to increase calcium absorption in patients with osteoporosis (207). *Akkermansia muciniphila* promotes intestinal calcium absorption and upregulates TRPV6 (a calcium transporter), thereby promoting bone mineralization (35). *In vivo*, high fiber diet particularly with resistant starch promotes propionate production, IL-10 and expansion of Treg cells in rheumatoid arthritis (208). In addition, while prebiotics and SCFAs exert stimulatory effects on intestinal calcium absorption, minerals, including copper, zinc, and iron, exert inhibitory effects (209). Further, exopolysaccharides from *B. longum* promote IL-10 expression in osteoclast precursors via TLR2 and inhibit osteoclast formation, thereby ameliorating osteoporosis (210).

## 4 Therapeutic targeting of the gut-immune-bone axis

### 4.1 Targeted prebiotic, probiotic, and postbiotic supplementation

Several pieces of evidence point towards the potential targeting of the complete gut-immune-bone axis in maintaining bone homeostasis and the management of bone diseases (54, 211). Evidence also suggests that a substantial portion of bone diseases is either influenced, caused, or driven by metabolic disruption (212, 213). Hence, targeting the rising metabolic dysregulation and related bone disorders through prebiotic, probiotic, and postbiotic supplementation is a promising strategy (214). **Table 1** summarizes the clinical evidence of these supplements against metabolic diseases and their impact on bone health parameters.

**Table 1:** Probiotic and prebiotic intervention against metabolic and bone diseases targeting the gut-bone axis: a consolidated clinical trial^#^.

Study design	Sample size & duration	Type of intervention and composition	Bone-related outcomes	Metabolic and systemic outcomes	Effect size* and direction of change	Ref.
Placebo-controlled, double-blind RCT; Patients with knee osteoarthritis	N=461; 6 mo	Probiotic; Skimmed milk with *Lactobacillus casei* Shirota (6 x 10^9^ CFU) 100ml/twice/day	Not assessed	WOMAC, VAS and CRP levels Reduced WOMAC and VAS scores and reduced hs-CRP	d = -1.42 (95% CI: -1.63 to -1.21) ↓ WOMAC	(215)
Placebo-controlled double-blind RCT; Early postmenopausal women	N=239; 2 yr	Probiotic; Limosilactobacillus reuteri 6475 Low dose (5 x 10^8^ CFU) and high dose (5 x 10^9^ CFU) 2 capsules/day	DXA and bone turnover markers	BMI, plasma SCFA, vitamin D and calcium No significant changes but modulated BMI	NR	(216)
Prospective multi-center, double-blind, placebo-controlled RCT; Postmenopausal women	N=221; 1 yr	Synbiotic SBD111 *L. brevis*, *L plantarum*, *L. mesenteroides*, *P. kudriazevii*, oligofructose and dried blueberry powder (4.75 x 10^10^ CFU) 2 capsules/day	DXA and bone turnover markers Reduced total hip and femoral neck BMD loss, although not significant	Serum inflammatory markers and microbiota composition – No significant changes	d = 0.06 (95% CI: -0.20 to 0.32) ↑ Total hip BMD d = 0.05 (95% CI: -0.20 to 0.32) ↑ Femoral neck BMD	(217)
Multicenter placebo-controlled RCT; Postmenopausal women	N=172; 12 mo	Probiotic Lacticaseibacillus (L.) paracasei (LPC100) and Lactiplantibacillus (L.) plantarum (LP140) 5 x 10^9^ CFU each 1 capsule/day	BMD Reduced BMD loss by improving T score	BMI, ALP, CRP, Serum vitamin D, calcium and phosphorous Reduced vitamin D deficiency	d = 0.18 (95% CI: -0.11 to 0.48) ↑ - T score	(218)
Multicenter, double blind, placebo controlled, parallel arm RCT; Hypercholesterolemic adults;	N=127; 9 wk	Probiotic, Lactobacillus reuteri 30242	Not assessed	LDL-cholesterol and vitamin D Reduced LDL-cholesterol and increased serum vitamin D	d = 0.35 (95% CI: 0.01 to 0.71) ↑- Vitamin D	(219)
Parallel design, double blind, placebo-controlled RCT; Postmenopausal women with osteopenia	N=78; 12 mo	Probiotic; Lactic acid probiotic and isoflavone aglycones (60mg) 2 sachets/day	DXA, bone turnover markers (CTX, P1NP) Reduced loss of BMD at L2-L4, trochanter and femoral neck, reduced CTX	Not assessed	d = -0.22 (95% CI: -0.67 to 0.21) ↓- CTX	(220)
Placebo controlled double blind RCT; Postmenopausal women	N=76; 24 wk	Probiotic; Bacillus subtilis C-3102 3 tablets/day Total dose of 3.4 x 10^9^ CFU	Bone resorption markers (uNTx, BAP), DXA Increased total hip BMD and reduced bone resorption (uNTx)	Microbiome analysis Promoted Bifidobacterium abundance	d = 2.94 (95% CI: 2.22 to 3.67) ↑- Total hip BMD d = -3.55 (95% CI: -4.35 to -2.74) ↓ - uNTx d = 2.32 (95% CI: 1.67 to 2.97) ↑- Bifidobacterium	(221)
Double-blind, placebo-controlled, parallel, multicenter RCT; Postmenopausal women;	N=72; 48 wk	Probiotic; L. brevis HA-112 and L. paracasei HA-274 (7.5 x 10^9^ CFU) 1 capsule/day	DXA and bone turnover markers (P1NP, BAP, CTX and osteocalcin) No significant changes compared to placebo	Not assessed	NR	(222)
Double-blind placebo-controlled RCT; Postmenopausal women aged 75-80years with low BMD;	N=70; 12 mo	Probiotic; Lactobacillus reuteri 6475 2 capsules/day (5 x 10^9^ CFU each)	Serum BAP, DXA Reduced loss of tibia total volumetric BMD	Not assessed	d = 1.02 (95% CI: 0.02 to 2.03) ↑- Tibia total vBMD	(223)
Quasi-experimental study; Postmenopausal women	N=54; 3 mo	Probiotic; Soymilk with honey fermented with *L. casei* R-68 or *L. plantarum* 1 R 1.3.2 100ml/day	Serum osteocalcin L. plantarum reduced osteocalcin levels	Cholesterol and blood glucose – No significant changes	d = -0.38 (95% CI: -1.04 to 0.27) ↓ - Osteocalcin	(224)
Double blind, placebo-controlled RCT; Postmenopausal women with osteopenia	N=50; 6 mo	Probiotic; Multispecies (L. casei, L. acidophilus, L. bulgaricus, L. rhamnosus, B. breve, B. longum, S. thermophilus) (500mg) 1 capsule/day	DXA, bone turnover markers (CTX, BAP, OC) Decreased BAP and CTX; No change in BMD	Serum PTH and inflammatory cytokines (TNF-α, IL-1β) Reduced PTH and TNF-α	d = -1.87 (95% CI: -2.61 to -1.14) ↓ - BAP d = -0.77 (95% CI: -1.40 to -0.13) ↓ - CTX d = -0.27 (95% CI: -0.89 to 0.33) ↓ - TNF-α d = -0.50 (95% CI: -1.13 to 0.11) ↓ - PTH	(225)
Double-blind, placebo-controlled RCT; Postmenopausal women with osteopenia	N=40; 12 wk	Synbiotic; Multispecies probiotic *L. reuteri* GL-104 (1.5 x 10^9^ CFU), *L. paracasei* MP-137 (6 x 10^8^ CFU), *L. rhamnosus* MP-108 (6 x 10^8^ CFU), *L. rhamnosus* F-1 (3 x 10^8^ CFU), *L. rhamnosus* BV77 (6 x 10^8^ CFU), *B. animalis* CP-9 (2.4 x 10^8^ CFU), *B. longum* OLP-01 (1 x 10^9^ CFU), *Bacillus coagulans* (1 x 10^9^ CFU) and Inulin (270mg) (1 sachet per day)	DXA, Bone resorption markers (CTX, P1NP) Reduced serum CTX in Synbiotic group	Not assessed	NR	(226)
Placebo-controlled RCT; Patients with postmenopausal osteoporosis	N=40; 3 mo	Probiotic; *Bifidobacterium lactis* (Probio-M8), calcium, calcitriol	BMD No significant changes	Microbiome analysis and serum vitamin D3, PTH and procalcitonin Increased SCFA producing bacteria, increased D3, reduced PTH and procalcitonin	NR	(106)
Crossover double-blind RCT; Postmenopausal women	N=20; 1d intervention with 1 wk washout	Probiotic; *Lactobacillus helvictus* fermented milk (220ml)	Not assessed	Serum calcium, ionized calcium PTH and phosphate Reduced serum PTH and improved serum calcium	NR	(227)
Placebo-controlled RCT; Postmenopausal women with low BMD	N=20; 1 yr	Probiotic; Lactobacillus reuteri ATCC PTA 6475	HR-pQCT, and bone turnover markers Increased BMD	Microbiome analysis and inflammatory markers Improved gene richness of the gut microbiota, reduced inflammation	NR	(228)
Double-blind RCT; Pubertal adolescents	N=95; 1 yr	Prebiotic; Short-chain and long-chain inulin type fructan (8g/day)	BMD, BMC Improved whole body BMC, BMD	Calcium - Increased calcium absorption	d = 0.48 (95% CI: 0.07 to 0.89) ↑- BMC d = 0.54 (95% CI: 0.13 to 0.95) ↑- BMD d = 0.40 (95% CI: -0.01 to 0.81) ↑- Calcium absorption	(229)
Placebo-controlled RCT; Adults with co-morbid obesity and knee osteoarthritis	N=54; 6 mo	Prebiotic; Oligofructose-enriched inulin (16g/day)	Not assessed	Performance based tests, knee pain, quality of life, microbiome analysis and serum metabolomics Increased physical performance, reduced fat mass, knee pain, Increased bifidobacterium abundance and promoted phenylalanine and tyrosine metabolism	NR	(230)
Crossover, double-blind placebo-controlled RCT; Diabetic adults	N=35; 6 wk	Prebiotic; Inulin type fructan (16g/day)	Bone turnover markers No significant changes; Correlation between bifidobacterium and P1NP	SCFA, serum vitamin and minerals Correlation between SCFA and vitamin D	NR	(231)
3-phase, double-blind crossover RCT; Healthy adolescent females	N=28; 4 wk	Prebiotic; Soluble corn fiber (0g/day, 10g/day; 20g/day)	Not assessed	Calcium absorption (ICP-MS), microbiome composition analysis Increased calcium absorption, promoted abundance of Parabacteroides and Clostridium	d = 0.33 (95% CI: -0.21 to 0.88) ↑- Calcium absorption d = 1.18 (95% CI: 0.59 to 1.77) ↑- Parabacteroides abundance	(232)
Double-blind, crossover RCT; Healthy adolescents	N=24; 6 wk	Prebiotic; Soluble maize fiber (12g/day)	Not assessed	Dual-stable isotopic measurement of fractional calcium and microflora composition Increased fractional calcium absorption by 12%; promoted abundance of Bacteroidetes	d = 0.11 (95% CI: -0.50 to 0.73) ↑- Fractional calcium absorption	(233)
Double-blind, crossover, placebo-controlled RCT; Postmenopausal women	N=18; 50d supplementation and 50d washout (thrice)	Prebiotic; Soluble corn fiber (0g/day, 10g/day and 20g/day)	DXA, Bone turnover markers (BAP, uNTx) and Bone calcium Increased BAP, improved bone retention of calcium by 4.8%	Not assessed	NR	(234)

^#^ Studies have been arranged in descending order of sample size; * For studies where effect sizes were not provided but sufficient data were available (mean, standard deviation and sample size), Cohen’s d was derived using the reported statistical values and represented as “Cohen’s d with 95% confidence interval”. The formula for effect size calculation in parallel group studies including quasi-experimental studies was: Cohen’ s $d=\frac{{\bar{x}}_{1}-{\bar{x}}_{2}}{S_{pooled}}$; (difference between group means divided by pooled standard deviation) while for non-parallel or crossover trials involving repeated measures, Cohen’s $d=\frac{{\bar{\Delta }}_{1}-{\bar{\Delta }}_{2}}{S_{pooled}}$ was used (pre-intervention (baseline) and post-intervention differences reported between intervention and control periods divided by pooled standard deviation). Studies lacking sufficient data were not included in effect size calculations and have been indicated as “NR” (not reported); ↑ - increased; ↓ - decreased; yr, year; mo, month; wk, week; d, day; RCT, Randomized controlled trial; CFU, Colony forming units; WOMAC, western Ontario and McMaster Universities Osteoarthritis index; VAS, Visual analogue scale; CRP, C-reactive protein; DASH, Disabilities of the arm; shoulder and hand; CRPS, Continuous ranked probability score; NRS, Numeric rating scale; SMI, Sustained maximal inspiration; MHQ, Michigan hand outcomes questionnaire; DXA, Dual energy X-ray absorptiometry; BMI, Body mass index; SCFA, Short chain fatty acids; BMD, Bone mineral density; ALP, Alkaline phosphatase; LDL, Low density lipoprotein; CTX, C-terminal telopeptide; P1NP, Procollagen type 1 amino terminal propeptide; uNTx, Urinary N-telopeptide; BAP, Bone-specific alkaline phosphatase; TNF-α, Tumor necrosis factor alpha; IL-1β, Interleukin 1 beta; PTH, Parathyroid hormone; HbA1c, Hemoglobin A1c; HR-pQCT, High resolution peripheral quantitative computed tomography; BMC, Bone mineral content; HOMA-IR, Homeostatic model assessment of insulin resistance; IL-6, Interleukin 6; MDA, Malondialdehyde; IL-4, Interleukin 4; IL-12, Interleukin 12; IFN-γ, Interferon gamma; ICP-MS, Inductively coupled plasma mass spectrometry.

Prebiotic fibers have been shown to promote intestinal fermentation, which is associated with increased SCFA production, increased calcium absorption, and increased bone density (235). Clinical evidence suggests that supplementation with soluble corn fiber significantly increased calcium absorption and bone alkaline phosphatase, a bone turnover marker (232). *In vivo*, lactose-derived galactooligosaccharides increased the abundance of Bifidobacteria, absorption of calcium and magnesium, calcium uptake in the femur, and the trabecular volumetric bone mineral density (236). In low doses, lactulose enhances mineral absorption and promotes the abundance of beneficial bacteria and metabolites (237). In a rat model of postmenopausal osteoporosis, polydextrose-based prebiotic enhanced calcium and magnesium absorption by improving SCFA production (238). Polysaccharides derived from *Sporidiobolus pararoseus* could alleviate rheumatoid arthritis by modulating arachidonic acid metabolism, OPG/RANKL/TRAF6 signaling, and osteogenic remodeling (239). Prebiotics also modulate the metabolomic profile and promote the expression of barrier proteins, thereby alleviating OA-related inflammation and cartilage degradation (240). Further, Lactobacillus acidophilus-fermented Astragalus polysaccharides show symbiotic effects on improving calcium absorption by altering metabolites in an osteoporosis model (241). Polysaccharides purified from *Eucommia ulmoides* oliver cortex increase bone mineralization, modulate osteoblast/osteoclast levels and mitigate osteoporosis (242). Dietary intervention with oligosaccharides from Konjac promoted calcium homeostasis, enhanced bone thickness and strength in calcium-deficient mice while also restoring Treg/Th17 balance in osteoporosis (243, 244).

Both preclinical and human models show the potential of probiotics to promote intestinal mineral absorption, increase mineral bioavailability, enhance bone mineralization, and minimize bone resorption (245–247). Probiotic supplementation shows potential beneficial effects on the microarchitecture of the jawbone in HFD-fed rats via the amelioration of osteoclast-related bone resorption (248). Supplementation with various probiotics, *Bacillus clausii, Lactobacillus rhamnosus, and B. longum*, attenuated bone loss by skewing the Treg/Th17 equilibrium in OVX mice (249–251). Fermented dairy products fortified with Lactobacillus acidophilus DSM 13241 were found to improve bone strength, increase SCFA production, improve barrier integrity, and increase the daily intake of phosphorus, magnesium, and calcium (252). *Lacticaseibacillus paracasei* LC86 could enhance bone and cartilage health, alter microbial profile, and promote anti-inflammatory pathways in osteoporosis model (253). In HFD-fed obese mice, probiotic supplementation improved osteoblast mineralization and bone formation via histone methylation (254). *Bifidobacterium longum* has been shown to modulate the intestinal microflora and increase serum vitamin D levels, thereby alleviating osteoporosis (105). Further, *C. butyricum* could mitigate bone loss, promote Th17/Treg, and balance osteoclastogenesis and osteoblast activity (255).

Postbiotics defined by ISAPP (International Scientific Association for Probiotics and Prebiotics) as “preparations of inanimate microbial cells and/or components that confer health benefits on the host” (256) are emerging as a promising therapeutic modality. Postbiotics also play roles in immune response regulation, antioxidant activity, microbial modulation, enhance barrier integrity, and show promise in the management of respiratory conditions, gastrointestinal disorders, and metabolic disorders (257). Interventions described as postbiotics or related products were classified into three major categories – (a) true postbiotics (conforming to ISAPP definition), (b) microbiota-based metabolites and (c) metabolite-based therapeutics. Different postbiotics conferring to the ISAPP definition have shown therapeutic effects against bone and metabolic diseases. Extract of *L. plantarum* L-14 could inhibit adipogenesis and improve obesity *in vivo* (258). Current preclinical studies show preliminary evidence of the beneficial effects of various postbiotic supplements in bone diseases. Postbiotics derived from *L. plantarum* BX 62 effectively reduce paw edema, arthritic damage, cytokine imbalances, and restore gut microbiota composition (259). *L. plantarum* MD35 protected against bone loss in OVX mice and modulated RANKL-mediated osteoclast differentiation (260). In other similar studies, heat-killed *L. reuteri* 6475, heat-inactivated *L. casei* GKC1, *L acidophilus*, *L. reuteri* and *B. longum* ameliorated bone loss and inhibited RANKL-induced differentiation of osteoclasts (261–263). Despite a few clinical studies highlighting the potential of postbiotic supplementation in prediabetic and diabetic patients with obesity (264, 265), there is a lack of clinical evidence on the impact of postbiotics on bone health.

In addition, microbiota derived metabolites such as exopolysaccharides derived from *Bifidobacterium breve* promoted butyrate producing bacteria and reduced HbA1c in prediabetic adults (266). Urolithin A, a microbiota-derived metabolite, reduced arthritis scores, inflammation, cartilage and bone destruction, and NF-κB pathway activation in a collagen-induced RA model (267). Further, Urolithin A also improved insulin sensitivity and attenuated triglyceride accumulation, potentially via augmentation of mitochondrial biogenesis (268). Cell free culture supernatant of *L. curvatus* Wikim38 has been observed to ameliorate bone loss in OVX mice (269). Exogenous polyamines also exhibit osteogenic effects, prevent bone loss, and stimulate bone formation (270, 271). Specifically in rheumatoid arthritis, various supplements, including vitamins, tryptophan metabolites, and exopolysaccharides, modulate RA by influencing microbiota homeostasis and immune functions (272). Limited evidence was found on the effect of metabolite-based therapeutics on bone health. Colon-targeted nanoparticle-delivered butyric acid suppressed inflammatory macrophage activation, reduced inflammatory responses, and regulated microbiome composition in the alleviation of osteoporosis (273).

### 4.2 Current evidence for fecal microbiota transplantation in bone diseases

Since the microbiome’s role in bone health was established, fecal microbiota transplantation (FMT) has emerged as a promising strategy. FMT refers to the transplantation of microbiota from healthy donors to recipients undergoing gut dysbiosis. Colonization by these microbial strains helps improve the gut barrier, reduce inflammation, and potentially modulate several intestinal and non-intestinal diseases. Even in conditions involving metabolic dysregulation and associated diseases, FMT has shown positive outcomes in both preclinical (274–276) and clinical settings (277).

Recent preclinical evidence further highlights the potential mechanisms of FMT in mitigating bone loss via improving barrier function and microbial composition (278, 279). FMT alleviated femoral head osteonecrosis by mitigating low butyric acid levels (280). Transplanted microbiota was found to reduce systemic inflammation and increase resistance to fracture in mice by altering mineral composition (281). In an LPS-induced osteoporosis model, FMT increased osteoblast levels, improved bone structure, and increased the abundance of Bacteroides and Lactobacillus, thereby alleviating OP (282). Again, transplantation of microbiota from OVX mice induced osteoporosis in healthy mice, suggesting the transfer of disease-associated metabolic signatures in addition to the microbiota (283). In the ACLT-induced osteoarthritis rat model, FMT from osteoarthritis patients further aggravated OA by elevating acetic acid, IL-6, and TNF-α, promoting synovitis, and causing severe cartilage degeneration (284). Further, FMT in mice from human donors with postmenopausal osteoporosis upregulated IL-17 and TNF-α and accelerated bone mass loss (285). **Table 2** summarizes the current pre-clinical evidence of FMT in bone disorders. Despite an unmet clinical trial gap in this area, the current preclinical data provide mechanistic insights into potential inflammatory responses and metabolite signatures in bone health, and reinforce the need for therapeutic targeting of the gut-immune-bone axis.

**Table 2:** Intervention of fecal microbiota transplantation (FMT) targeting bone disorders: preclinical evidences.

Study objective	Type of FMT and delivery route	Animal strain, age sample size, duration	Parameters	Outcome	Ref.
Effect of mulberry polysaccharide treatment in ameliorating KOA using FMT	FMT from Crude mulberry polysaccharide treated donor rats to KOA rats; Oral administration	Male SD rats; 8 wk old; N = 20; 7 wks	Gait analysis, joint circumference, knee joint histopathology	↑ Gait parameters – stride length, footprint width ↓ Accumulation of hypertrophic chondrocytes, loss of cartilage matrix	(286)
Effect of FMT in osteoporosis (OVX-induced model)	FMT from healthy donor mice to OVX-treated mice; Oral administration	Female C57BL/6 mice; 8 wk old; N = 20; 9 wks	μCT, Femur histopathology, Intestinal barrier integrity, Cytokine expression	↑ BV/TV, BS/TV, BMD, Tb.Th, Tb.N ↑ expression of ZO-1, occludin ↓ TNF-α, IL-1β ↑ fecal SCFA levels – acetic acid, propionic acid	(278)
Effect of PEMF exposure in ameliorating HFD-induced metabolic and osteogenic dysfunction using FMT	FMT from PEMF-treated donor mice to HFD-mice; Oral administration	Male C57BL/6NTac mice; 7 wk old; N = 81; 16 wks	μCT, 16s rRNA sequencing	↓ F/B ratio ↑ cortical volume, cortical BMD, Tb.Th, Tb.N	(287)
Effect of FMT in osteoporosis (LPS-induced model)	FMT from healthy donors to LPS-induced OP mice; Oral administration	Male and female (1:1) C57BL/6 mice; 6 wk old N = 20; 3 wks	μCT, 16s rRNA sequencing	↑ RUNX2 expression in femoral sections, ↑ lncRNA TUG1 expression in blood and gut ↑ Alpha diversity (Chao 1 index)	(282)
Effect of FMT in senile osteoporosis	FMT from 3-mo healthy donor rats to aged (18-mo) rats; Oral administration	Female SD rats; 18 mo old; N = 36; 24 wks	DXA, μCT, 16s rRNA sequencing, serum bone turnover markers	↑ BV, BV/TV, Tb.Th, Tb.N ↓ P1NP, CTX	(279)
Effect of tuna oil supplementation in ameliorating RA using FMT	FMT from Tuna Oil treated CIA mice to CIA-induced mice; Oral administration	Male ICR mice; 5 wk old; N = 15; 10 wks	μCT, cytokine expression,	↓ TNF-α, IL-1β, IL-17A ↑ IL-10 ↓ Arthritis severity score	(288)

mo, month; wk, week; KOA, Knee osteoarthritis; FMT, Fecal microbiota transplantation; SD, Sprague Dawley; OVX, ovariectomy; μCT, Micro Computed Tomography; BV, Bone volume; BV/TV, Bone volume fraction; BS/TV, Bone surface per total volume; BMD, Bone mineral density; Tb.Th, Trabecular Thickness; Tb.N, Trabecular Number; ZO-1, Zonula occludens-1; TNF-α, Tumor necrosis factor alpha; IL-1β, Interleukin 1 beta; SCFA, Short chain fatty acids; PEMF, Pulsed electromagnetic field; HFD, High fat diet; F/B, Firmicutes to Bacteroides ratio; LPS, Lipopolysaccharide; OP, Osteoporosis; RUNX2 - Runt-related transcription factor 2; lncRNA, Long non-coding ribonucleic acid; TUG1, Taurine upregulated gene 1; DXA - Dual energy X-ray absorptiometry; P1NP, Procollagen type 1 amino terminal propeptide; CTX, C-terminal telopeptide; CIA, Collagen-induced arthritis; ICR, Institute of cancer research; IL-17A, Interleukin 17A; IL-10, Interleukin 10.

## 5 Conclusions

Gut dysbiosis, associated with altered production of gut metabolites, contributes to metabolic dysregulation. Various studies reported that the gut microbiota and their metabolites eventually regulate bone mass. Distinct microbial and metabolic profiles from clinical data, such as the enrichment of *Lachnospira eligens* in fracture patients with low bone mass, suggest a potential pathogenic role for specific microbial species. On the other hand, symbiotic gut microbes such as Bacteroides uniformis alleviate metabolic dysfunction by promoting bile acid production, including 3-succinylated cholic acid. Gut metabolites such as SCFAs, tryptophan metabolites, bile acids, and polyamines have been shown to be involved in bone remodeling by promoting osteoblastogenesis and inhibiting osteoclastogenesis in several *in vivo* studies. SCFAs are involved in whole-body metabolism, with diverse functions as signaling molecules and as key regulators of immunity, glucose, and lipid metabolism. The expression of SCFA receptors such as GPR41, GPR109A in osteoblast precursors and subsequent inhibition of osteoclast differentiation by propionate and butyrate suggest the potential modulation of bone resorption by SCFAs. Preclinical evidence suggests SCFAs promote bone growth, remodeling and reduce pathological bone loss via induction of IGF-1. Tryptophan and its metabolites play modulatory roles in osteochondral destruction associated with osteoporosis, osteoarthritis, and rheumatoid arthritis, indicating their therapeutic potential. Tryptophan metabolites such as IAA and IPA act as aryl hydrocarbon receptor ligands, activate M2 macrophage polarization, which releases IL-10 that inhibits osteoclastogenesis and promotes osteoblastogenesis during osteoporosis. *In vivo* studies suggest that elevated bile acid levels observed in osteoporosis are negatively correlated with bone mineral density. Excessive TMAO release accelerates osteoporosis by promoting bone loss and inhibiting osteogenic differentiation. For example, TMAO enhances RANK ligand-induced osteoclast differentiation and bone resorption by NF-κB activation. Particular microbial species, short-chain fatty acids, and other gut-derived factors also exhibit immunomodulatory properties. The emerging roles of T cells, B cells, and macrophages in immune regulation during bone remodeling underscore the potential of gut-mediated immune modulation to maintain bone health. Comprehensive effects of the gut microbiome, including gut metabolites, hormones, vitamins, and minerals, modulate bone homeostasis either directly or via immune regulation. Overall, the gut-immune-bone axis plays a crucial role in metabolic dysregulation associated with bone diseases.

The mediating roles of gut microbiota and their metabolites in the pathogenesis of various bone disorders are being considered, and thus, ameliorating these conditions by targeting intestinal and immune homeostasis could be beneficial. For example, fermented dairy products fortified with *Lactobacillus acidophilus* were found to promote intestinal mineral absorption and bioavailability, support bone mineralization, and minimize bone resorption in RCTs. Evidence from *in vivo* studies indicates that polyamines and SCFAs can maintain Th17/Treg homeostasis and inhibit osteoclastogenesis. Several clinical intervention trials with FMT target metabolic dysregulation and associated disorders. FMT proposed to mitigate bone loss in several *in vivo* studies by improving barrier function and microbial composition. Recent *in vivo* studies suggest that true postbiotics, microbiota-based metabolites and metabolite-derived therapeutics are emerging. However, clinical evidence of postbiotics or related products on bone health is needed.
